# Mellein: Production in culture by *Macrophomina phaseolina* isolates from soybean plants exhibiting symptoms of charcoal rot and its role in pathology

**DOI:** 10.3389/fpls.2023.1105590

**Published:** 2023-02-08

**Authors:** Vivek H. Khambhati, Hamed K. Abbas, Michael Sulyok, Maria Tomaso-Peterson, Jian Chen, Wayne Thomas Shier

**Affiliations:** ^1^ Department of Biochemistry, Molecular Biology, Entomology, and Plant Pathology, Mississippi State University, Mississippi State, MS, United States; ^2^ Biological Control of Pests Research Unit, Biological Control, United States Department of Agriculture – Agricultural Research Service, Stoneville, MS, United States; ^3^ Department of Agrobiotechnology (IFA–Tulln), Institute of Bioanalytics and Agro-Metabolomics, University of Natural Resources and Life Sciences Vienna, Tulln, Austria; ^4^ Department of Medicinal Chemistry, College of Pharmacy, University of Minnesota, Minneapolis, MN, United States

**Keywords:** mycotoxin, natural product, phytotoxin, bioassay, chemical analysis, fungi, hydroponic assay

## Abstract

*Macrophomina phaseolina* (*Mp*) is a fungal pathogen proposed to enter host roots by releasing toxins that induce local necrosis in roots allowing entry of hyphae. *Mp* is reported to produce several potent phytotoxins, including (-)-botryodiplodin and phaseolinone, but isolates that do not produce these phytotoxins retain virulence. One hypothesis explaining these observations is that some *Mp* isolates may produce other unidentified phytotoxin(s) responsible for virulence. A previous study of *Mp* isolates from soybean found 14 previously unreported secondary metabolites using LC-MS/MS, including mellein, which has various reported biological activities. This study was conducted to investigate the frequency and amounts of mellein produced in culture by *Mp* isolates from soybean plants exhibiting symptoms of charcoal rot and to investigate the role of mellein in any observed phytotoxicity. LC-MS/MS analysis of cell-free culture filtrates (CCFs) from 89 *Mp* isolates revealed that 28.1% produced mellein (49–2,203 µg/L). In soybean seedlings in hydroponic culture, *Mp* CCFs diluted to 25% (vol/vol) in hydroponic growth medium induced phytotoxic symptoms with frequencies of 73% chlorosis, 78% necrosis, 7% wilting, and 16% death, and at 50% (vol/vol) induced phytotoxicity with frequencies of 61% chlorosis, 82% necrosis, 9% wilting, and 26% death. Commercially-available mellein (40–100 µg/mL) in hydroponic culture medium induced wilting. However, mellein concentrations in CCFs exhibited only weak, negative, insignificant correlations with phytotoxicity measures in soybean seedlings, suggesting that mellein does not contribute substantially to observed phytotoxic effects. Further investigation is needed to determine if mellein plays any role in root infection.

## 1 Introduction

The fungus *Macrophomina phaseolina* (Tassi) Goid. is an opportunistic soil-borne and seed-borne pathogen found worldwide that infects over 500 plant species including row crops, fruits, trees, and ornamentals (Su et al., 2001; Hartman et al., 2015; Ghosh et al., 2018). The phytopathogen targets the stem and root systems of plants and spreads through the soil rhizosphere by interconnected root systems (Meyer et al., 1974; Pearson et al., 1984). *M. phaseolina* can survive as a saprophyte in soil between growing seasons, and microsclerotia produced by the fungus can persist in plant debris and soil for up to 10 years (Short et al., 1980; Ghosh et al., 2018). In the US, *M. phaseolina* is known for causing charcoal rot of soybean (*Glycine max* L.) and, when left unmanaged, can result in significant economic losses for farmers by decreasing yields and seed quality of soybean (Bowen and Schapaugh, 1989; Wrather and Koenning, 2006; Gupta et al., 2012; Allen et al., 2017; Heatherly, 2020). Disease occurrence and severity is more prevalent in the southern US where conditions such as high temperatures and dry weather along with poor crop management can create drought-stressed plants which are more susceptible to infection by *M. phaseolina* and other pathogens (Kendig et al., 2000; Gupta et al., 2012). This prevalence and severity of charcoal rot may be attributed to the genetic variability of *M. phaseolina* and the diversity of mycotoxins produced by the pathogen (Dhingra and Sinclair, 1972; Jana et al., 2005; Reyes-Franco et al., 2006).

Previous studies have reported that *M. phaseolina* isolates produce a variety of mycotoxins including asperlin, phomalactone, phaseolinic acid, phaseolinone, and (-)-botryodiplodin (Dhar et al., 1982; Mahato et al., 1987; Bhattacharya et al., 1992; Ramezani et al., 2007), but production of these mycotoxins appears to be inconsistent across hosts and regions (Ramezani et al., 2007; Lodha and Mawar, 2020). For instance, Dhar et al. (1982) reported that *M. phaseolina* isolated as an endophyte from mung bean in India produced phaseolinone, but they did not report the presence of (-)-botryodiplodin, whereas Ramezani et al. (2007) reported that *M. phaseolina* isolated from a soybean plant exhibiting symptoms of charcoal rot in Mississippi, USA, produced (-)-botryodiplodin, but production of phaseolinone could not be detected. *M. phaseolina* has been proposed to infect hosts through a toxin-mediated process in which the toxin creates a readily penetrated necrotic area in the root, but the process is not well understood (Bellaloui et al., 2012; Abbas et al., 2019; Abbas et al., 2020). Analysis of secondary metabolites produced by *M. phaseolina* isolates was a necessary step in identifying pathogenic capability and characterizing pathogenic mechanisms in the disease. Genetic diversity among fungal isolates enables production of multiple secondary metabolites with the possibility of more than one root infection mechanism and regional variations are expected to contribute to the genetic variability (Reyes-Franco et al., 2006; Sarr et al., 2014).

Regional variability in secondary metabolite production may explain why Khambhati et al. (2020a) discovered mellein production by *M. phaseolina* isolates from soybean by GC-MS analysis using the freeze-thaw method (Chen, 2017). Later, Salvatore et al. (2020) independently confirmed the production of mellein by *M. phaseolina* isolated from *Eucalyptus globus*. Further work by Khambhati et al. (2020b) used LC-MS/MS to identify an additional 13 previously unreported metabolites produced in culture by *M. phaseolina* isolates from soybean plants exhibiting symptoms of charcoal rot. This study used LC-MS/MS to confirm mellein production in 28.1% of 89 *M. phaseolina* isolates. Additional studies using LC-MS/MS by Alam et al. (2022) reported production in culture of two metabolites not previously reported to be produced by *M. phaseolina*, cyclo(L-Pro-L-Val) and dihydrocitrinone, but they did not detect production of mellein.

Mellein, a dihydroisocoumarin compound, was first reported to be produced by *Aspergillus melleus* Yukawa in 1933 (Nishikawa, 1933) and later was reported to be produced by *A. ochraceus* K. Wilhelm in association with ochratoxin A, which is where the compound receives the alternative name, ochracin (Moore et al., 1972). However, the compound was found not to play an intermediatory role in the biosynthesis of ochratoxin A despite structural similarity (Harris and Mantle, 2001). Mellein is produced by several different fungi, including *Botryosphaeria obtusa* (Schweinitz) Shoemaker [syn. *Diplodia seriata* De Notaris] (Djoukeng et al., 2009), *Lasiodiplodia theobromae* (Pat.) Griffon & Maubl. (Aldridge et al., 1971), *Septoria nodorum* (Berkeley) Berkeley [syn. *Parastagonospora nodorum* (Berk.) Quaedvlieg, Verkley & Crous] (Sachse, 1992) and many others (Reveglia et al., 2020), as well as plants, insects, and bacteria (Brand et al., 1973; Harris and Mantle, 2001; Chen, 2017; Reveglia et al., 2020). Mellein is a biologically active compound and is toxic to plants, cell, fungi, algae, and insect larva (Nishikawa, 1933; Aldridge et al., 1971; Moore et al., 1972; Brand et al., 1973; Sachse, 1992; Harris and Mantle, 2001; Cabras et al., 2006; Djoukeng et al., 2009; Citron et al., 2012; Reveglia et al., 2020).

Of the new *M. phaseolina* metabolites identified by LC-MS/MS, mellein is very significant as it is a known compound with multiple reports of phytotoxic, cytotoxic, fungicidal, antibacterial, and larvicidal activity (Reveglia et al., 2020). The identification of all secondary metabolites produced by *M. phaseolina* will help to better understand the pathogenesis of *M. phaseolina* (Bellaloui et al., 2012; Abbas et al., 2019; Abbas et al., 2020). Examples of phytotoxic responses reported to be induced by mellein include inducing necrotic leaf spots in hemp dogbane (*Apocynum cannabinum* L.) (Venkatasubbaiah et al., 1992) and other plants (Reveglia et al., 2020), as well as inducing necrotic spots on apple (*Malus domestica*) fruits and stems and causing necrotic spots, browning and leaf necrosis in apple seedlings cultured in (R)-(-)-mellein solution at 150 mg/L (Li et al., 2021). Presently there are no reported studies on the phytotoxicity of mellein in a soybean system, so the objectives of this study were to evaluate a collection of *M. phaseolina* isolates from plants exhibiting symptoms of charcoal rot for mellein production in cell-free culture filtrates (CCFs), to evaluate the phytotoxic effects of *M. phaseolina* CCFs from the same isolates exposed to soybean seedling roots in hydroponic culture and to compare CCF phytotoxicity to the phytotoxic effects of commercially-available mellein under the same conditions.

## 2 Materials and methods

### 2.1 Fungal cultures and sources

A total of 89 *M. phaseolina* isolates were collected and used in this study (**Table 1**). All cultures were provided by other investigators and isolated using the method described by Mengistu et al. (2007) from charcoal rot-infected soybean plants, which were collected from various fields in Illinois, Louisiana, Mississippi, Oklahoma, and Tennessee. Cultures were maintained on potato dextrose agar (Becton, Dickinson and Company, Sparks, MD, USA) and stored at 4°C until use.

**Table 1:** Phytotoxicity of cell-free culture filtrates prepared with the indicated *Macrophomina phaseolina* isolates against soybean seedlings of germplasm line DT97-4290 in hydroponic culture.

			Filtrate concentration in hydroponic culture medium, 50%/25% (vol/vol)^z^					
Isolate	Collection Site^y^	Mellein (µg/L)	Phytotoxicity Rating	Dry Weight	Stem Growth	Volume Consumed	Wilting	Death
A-2	MS	0	NS/NS	NS/NS	NS/NS	NS/NS	-/-	-/-
A-3	MS	0	NS/NS	NS/NS	NS/NS	NS/NS	-/-	-/-
A-4	MS	1708	NS/NS	NS/NS	NS/NS	NS/NS	-/-	-/-
A-5	MS	0	NS/NS	NS/NS	NS/NS	+/NS	-/-	-/-
A-6	MS	0	NS/++	NS/NS	NS/+	NS/+	-/+	-/+
A-7	MS	0	NS/+++	NS/NS	NS/+	+/+	+/-	+/+
A-8	MS	0	NS/NS	NS/NS	NS/+	NS/NS	-/-	-/-
A-9	MS	0	NS/++	+/NS	NS/NS	+/+	-/-	-/+
A-10	MS	0	+/++++	NS/NS	NS/++	NS/++++	-/-	-/+
A-11	MS	581.7	NS/+	NS/NS	NS/+	NS/+	-/-	-/-
A-12	MS	309.1	NS/NS	NS/NS	NS/NS	NS/NS	+/-	-/-
A-13	MS	170.2	NS/NS	NS/NS	NS/NS	NS/++	-/-	-/-
A-14	MS	0	NS/NS	NS/NS	NS/NS	NS/NS	-/-	-/-
A-15	MS	0	NS/NS	NS/NS	NS/NS	NS/NS	-/-	-/-
A-16	MS	79.72	NS/NS	NS/NS	NS/NS	NS/NS	-/-	-/-
A-17	MS	678.7	NS/NS	NS/NS	NS/NS	NS/NS	-/-	-/-
A-18	MS	50.29	++/++	NS/NS	NS/NS	NS/NS	-/-	-/+
A-19	MS	356.2	+/NS	NS/NS	NS/NS	NS/NS	-/-	-/-
A-20	MS	97.99	+/NS	NS/NS	NS/NS	NS/+	-/-	-/-
A-21	MS	0	NS/NS	NS/NS	NS/NS	NS/NS	-/-	-/-
A-22	MS	0	++++/++++	NS/NS	NS/NS	NS/++++	-/-	+/+
A-23	MS	0	+++/++++	+/NS	NS/+	NS/NS	-/-	-/+
A-24	MS	48.87	NS/++++	NS/NS	NS/NS	NS/NS	-/-	-/-
A-25	MS	70.48	++++/++++	NS/+	NS/++	NS/+	-/-	+/+
A-26	MS	0	NS/++++	NS/NS	NS/NS	NS/NS	-/-	-/-
A-27	MS	0	NS/NS	NS/NS	NS/NS	NS/NS	-/-	-/-
A-28	MS	51.94	NS/+	NS/NS	NS/++	NS/++	-/-	-/-
A-29	MS	0	NS/NS	NS/NS	NS/NS	NS/NS	-/-	-/-
A-30	MS	0	NS/NS	NS/NS	NS/NS	NS/NS	-/-	-/-
A-31	MS	0	+/NS	NS/NS	NS/NS	NS/NS	-/-	-/-
A-32	MS	0	NS/NS	NS/NS	NS/NS	NS/NS	-/-	-/+
A-33	MS	0	NS/NS	NS/NS	NS/NS	NS/NS	-/-	-/-
A-34	MS	190.6	NS/++	+/NS	NS/++++	++/++	-/-	-/-
A-35	MS	0	++++/++++	++/++	+/++++	+++/++++	+/-	+/+
A-36	MS	120.1	+/++++	NS/+	NS/++++	++/+++	-/-	-/+
A-37	MS	0	++++/++++	++/++	+/++++	+++/+++	-/-	+/+
B-1	MS	0	++++/++++	++/NS	+/++++	++/+++	-/-	+/+
B-2	MS	341.5	++++/+++	+/NS	+/++++	++/+++	-/-	+/+
B-3	MS	0	+/NS	NS/NS	++/+++	NS/NS	-/-	-/-
B-4	MS	0	++++/+	+/NS	++/++++	NS/NS	-/-	-/-
C-1	MS	145.1	++++/+++	NS/NS	++/++++	NS/NS	-/-	+/+
C-2	MS	224.8	+++/NS	NS/NS	NS/++++	NS/NS	-/-	-/-
C-3	MS	52.74	++++/++++	++/NS	+/++++	NS/NS	-/-	+/+
C-4	MS	0	++++/+++	NS/NS	NS/++++	NS/NS	-/-	-/-
C-5	MS	349.4	NS/++++	NS/NS	++/NS	NS/NS	-/-	-/-
C-6	MS	0	++++/++++	+/+++	NS/NS	NS/+++	-/-	-/-
C-8	MS	0	++++/+++	NS/NS	NS/NS	+/++	-/-	-/-
C-9	MS	2203	+/+	NS/NS	NS/++	++/++	-/-	-/-
C-10	MS	884.8	++/++++	NS/+	NS/+	NS/NS	-/-	-/-
C-11	MS	0	+/++++	NS/NS	NS/++	NS/NS	+/+	-/-
C-12	MS	0	+++/++++	NS/+++	++++/++++	+++/++++	-/-	+/+
C-13	MS	228.3	++++/++++	NS/NS	NS/NS	NS/NS	-/+	+/-
C-14	MS	104.7	+/+	NS/NS	+/++++	NS/NS	-/-	-/-
C-15	MS	0	++/NS	NS/NS	+/++++	+++/++++	-/-	-/-
D-1	MS	0	+++/++++	NS/NS	NS/NS	NS/NS	-/-	-/-
D-2	MS	0	++++/++++	+/NS	++++/++++	+++/++++	+/+	-/-
D-3	MS	55.45	++/++++	NS/NS	NS/++	NS/NS	-/-	-/-
D-4	MS	97.73	+/++++	NS/++	+/++++	NS/++++	-/-	-/-
D-5	MS	0	++/++++	NS/++	++/++++	+/++++	+/+	-/-
D-6	MS	0	+++/NS	NS/+	++/++	NS/++	-/-	-/-
D-7	MS	0	++++/++++	++/NS	+/+	++++/+++	-/-	+/+
D-8	MS	0	++++/++++	NS/NS	+++/++++	++/++++	-/-	-/-
D-9	MS	0	++++/+++	NS/NS	NS/NS	NS/NS	-/-	-/-
D-10	MS	0	++/++++	NS/NS	NS/+	NS/NS	-/-	-/-
E-1	MS	0	++++/++	NS/NS	++++/++++	+/++	-/-	-/-
MP-235	MS	0	++++/++++	NS/++	NS/++	+/++++	-/-	-/-
TN-4	TN	0	NS/NS	+/+	NS/+	++++/++++	-/-	-/-
TN-260	TN	0	+++/+++	NS/+++	++++/+++	+++/+++	-/-	-/-
TN-261	TN	0	+++/++++	+/++++	++++/++++	+++/++++	-/-	-/+
TN-262	TN	0	++/++++	NS/++++	++++/++++	++/++++	-/-	-/-
TN-263	TN	0	+/++	NS/++	+/++	NS/++	-/-	-/-
TN-264	TN	0	++/++++	NS/++++	++++/++++	++/++++	-/-	-/+
TN-279	TN	0	+/++	NS/NS	++/++++	NS/++	-/+	-/-
TN-280	TN	0	+/+++	NS/NS	+++/++++	NS/++	-/+	-/-
TN-281	TN	0	++/+++	NS/NS	++++/++++	+/+++	-/-	-/+
TN-282	TN	0	+/NS	NS/NS	+++/++++	NS/++	-/-	-/-
TN-283	TN	0	+/NS	NS/NS	NS/+	NS/NS	-/-	-/-
TN-314	TN	0	NS/NS	NS/NS	+/+++	NS/NS	-/-	-/-
TN-315	TN	0	NS/NS	NS/NS	++/+	NS/NS	-/-	-/-
TN-316	TN	0	+++/++	NS/+	NS/++	+/++++	-/-	-/-
TN-317	TN	0	++++/++++	+/++	++/+++	++++/++++	-/-	+/+
TN-318	TN	0	+/NS	+/NS	+++/NS	+/NS	-/-	-/-
TN-319	TN	0	NS/+	NS/NS	+/+++	+/++++	-/-	-/-
TN-320	TN	0	NS/NS	NS/NS	NS/+	NS/+++	-/-	-/-
TN-321	TN	0	++/+	+/+	NS/++++	+++/++++	-/-	-/-
TN-518	TN	0	+/NS	NS/+	+/++++	++/++++	-/-	-/-
MP-205	LA	0	NS/++	NS/NS	NS/NS	NS/++	-/+	-/-
MP-279	OK	0	++++/++++	+/++	++/++	++/+++	-/+	-/-
U18	IL	0	++++/+	NS/NS	NS/NS	NS/NS	-/-	-/-

^y^ IL, Illinois, USA; LA, Louisiana, USA; MS, Mississippi, USA; OK, Oklahoma, USA; TN, Tennessee, USA. ^z^ Significant differences relative to the control are depicted with ++++ for differences at the *p* < 0.0001 level of significance, as +++ for *p* < 0.001, as ++ *p* < 0.01 and as + for *p* < 0.05. NS denotes no statistical significance.

### 2.2 Chemicals and reagents

Sucrose, sodium nitrate, potassium chloride, dipotassium phosphate, magnesium sulfate, iron (II) sulfate, and agar were purchased from Fisher Chemicals (Fair Lawn, NJ, USA). Enantiomerically pure (R)-(-)-mellein purchased from Cayman Chemical (Ann Arbor, MI, USA) with a listed purity of ≥98%, which was confirmed by GC-MS analysis (Chen, 2017), was used as an LC-MS/MS standard and for phytotoxicity testing with soybean seedlings. 3-Nitropropionic acid for use as an LC-MS/MS standard was purchased from Sigma-Aldrich (Vienna, Austria). For culture medium preparation and phytotoxicity assays, reverse osmosis water was purified by a Barnstead E-Pure Ultrapure Water Purification System (Thermo Scientific, Marietta, OH, USA).

Reagents for LC-MS/MS analysis consisted of LC-MS grade ammonium acetate and glacial acetic acid purchased from Sigma-Aldrich (Vienna, Austria), LC-MS Chromasolv grade methanol purchased from Honeywell (Seelze, Germany), HiPerSolv Chromanorm HPLC gradient grade acetonitrile purchased from VWR Chemicals (Vienna, Austria), and reverse osmosis water purified using a Purelab Ultra system (ELGA LabWater, Celle, Germany).

### 2.3 LC-MS/MS analysis

An extension of the LC-MS/MS method defined by Sulyok et al. (2020) was followed to analyze samples for an extended set of metabolites including mellein. Compound-specific LC-MS/MS parameters for mellein and 3-nitropropionic acid were determined *via* direct infusion of standards (diluted in a 1:1 mixture of eluent A and B) into the MS source using a syringe injection at a flow rate of 10 μL/min. Mellein and 3-nitropropionic acid were identified in column effluents after chromatographic separation using the retention times, precursor ion mass-to-charge ratio measurements before fragmentation, declustering potentials and mass-to-charge ratios, collision energy values, and cell exit potentials of diagnostic ions in the electron impact mass spectra of the fragmented precursor ions as given in **Table 2**. An Agilent 1290 series UHPLC system (Waldbronn, Germany) was coupled with a Sciex QTrap 5500 system (Foster City, CA, USA) fitted with a Sciex TurboV electrospray ionization source (Foster City, CA, USA). The settings of the ESI source were as follows: source temperature 550°C, curtain gas 30 psi (206.8 kPa of max. 99.5% nitrogen), ion source gas 1 (sheath gas) 80 psi (551.6 kPa of nitrogen), ion source gas 2 (drying gas) 80 psi (551.6 kPa of nitrogen), ion-spray voltage −4,500 V and +5,500 V, respectively, collision gas (nitrogen) medium. Analyte detection was accomplished by scheduled multiple reaction monitoring (sMRM), with a cycle time of 1.5 sec, a retention time window of 40 sec and a resolution of 0.7 amu for both Q1 and Q3. A Phenomenex (Torrance, CA, USA) Gemini C18 column (150 x 4.6 mm i.d. and 5 µm particle size) with a Phenomenex C18 security guard cartridge (4 x 3 mm i.d.) was attached for chromatographic separation. The system was run in binary gradient mode with a 1 mL/min flow rate and 5 µL injection volume. Both mobile phases consisted of methanol/water/acetic acid containing 5 mM ammonium acetate with Eluent A 10:89:1 (v/v/v) and Eluent B 97:2:1 (v/v/v). The gradient system was started by holding 100% A for 2 minutes followed by linearly increasing to 50% B over 3 minutes. The gradient was linearly increased to 100% B over 9 minutes and held at 100% B for 4 minutes. The run was completed by re-equilibrating back to 100% A within 2.5 minutes.

**Table 2:** Optimized LC-MS/MS parameters for mellein and 3-nitropropionic acid.

Compound	Retention Time (min)	Precursor Ion *m/z*	DP^a^	Fragment Ion *m/z*	CE^b^ (V)	CXP^c^ (V)
3-Nitropropionic acid	2.9	118	-65	46	-16	-3
Mellein	9.1	179.1	96	105.1	32	40
				115.1	8	8

^a^ DP, declustering potential, ^b^ CE, collision energy, ^c^ CXP, cell exit potential.

For unambiguous confirmation of the identity of the analytes, the criteria set in SANTE/12089/2016 have been applied with a stricter in-house criterion of a maximum tolerance of ± 0.03 min in retention time compared to an authentic standard. Quantification was based on external calibration using a serial dilution of a multi-analyte stock solution. As the culture filtrates did not induce any significant signal suppression/enhancement due to matrix effects, no correction was applied. The accuracy of the method is verified for major mycotoxins by participation in a proficiency testing scheme organized by BIPEA (Gennevilliers, France) with > 95% of the 1800 results submitted so far exhibiting a z-score of -2 < z < 2. As for the remaining metabolites, the large majority complied to performance criteria set for recovery of the extraction, repeatability, and within laboratory reproducibility (Sulyok et al., 2020). The related values for mellein determined in four processed grain-based matrices are 96 – 112% for the recovery of extraction, 5 – 10% for the repeatability, and 5-12% for the within laboratory reproducibility (Sulyok et al. unpublished data).

### 2.4 Hydroponic growth medium

Villagarcia medium is a nutrient growth medium suitable for hydroponic soybean seedling growth and was prepared using macronutrient and micronutrient solutions (Ahmed and Evans, 1960; McClure and Israel, 1979; Villagarcia et al., 2001). The macronutrient solution was prepared by adding 690 mg CaSO_4_·2H_2_O, 34 mg KH_2_PO_4_, 200 mg KNO_3_, 61 mg MgSO_4_·7H_2_O to 999 mL of sterile distilled water. The micronutrient solution was prepared in 1000-fold concentration by adding 50 mg FeSO_4_·7H_2_O, 14 mg KCl, 5.7 mg H_3_BO_4_, 1.5 mg MnSO_4_·H_2_O, 2.6 mg ZnSO_4_·7H_2_O, 0.45 mg CuSO_4_·5H_2_O, and 2.1 mg (NH_4_)_6_Mo_7_O_24_ to 1 L sterile distilled water. Then, 1 mL of the micronutrient solution was added to 999 mL of the macronutrient solution to form the 100% hydroponic medium.

### 2.5 Soybean plants and growth chamber conditions

**Figure 1:**
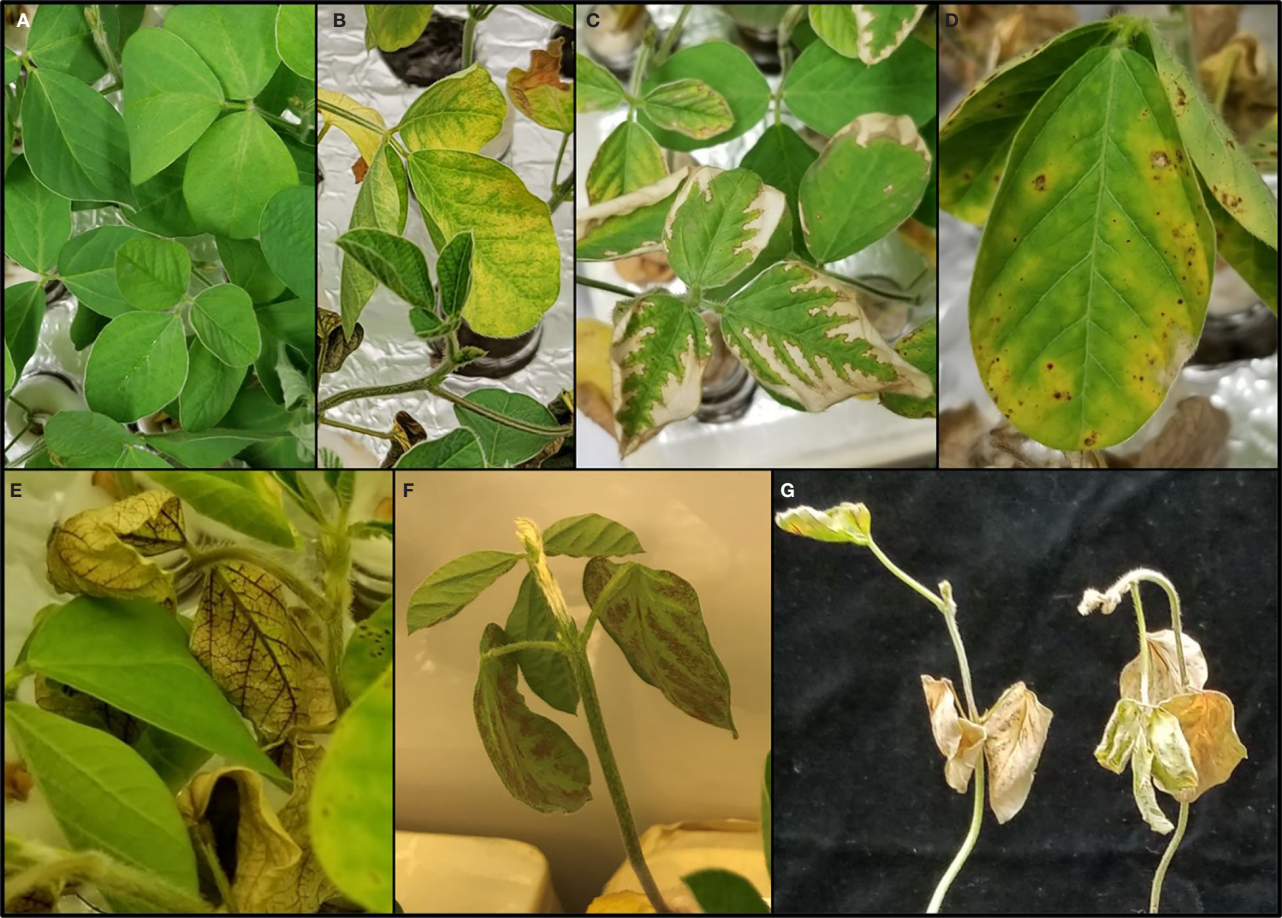
Effects of various toxins produced by *Macrophomina phaseolina* on soybean seedling foliage. The responses from the experiment ranged from no phytotoxic effect, as shown in the healthy plant control **(A)**, to the following phytotoxic responses: chlorosis **(B)**, photobleaching **(C)**, necrosis **(D)**, vascular discoloration **(E)**, intervascular discoloration **(F)**, and death **(G)**.

For this experiment, the soybean genotype DT97-4290 was selected for its moderate susceptibility to charcoal rot (Paris et al., 2006; Mengistu et al., 2011). Soybean seeds were surface disinfected by soaking seeds in 5% sodium hypochlorite solution for 2 minutes and rinsed several times with sterile water to remove any residue of sodium hypochlorite. Jiffy 7^®^ 42 mm peat pellets (Jiffy Products of America Inc., Lorain, OH, USA) were placed in a tray, and warm water was added to the tray to rehydrate the peat pellets. A single soybean seed was planted in each peat pellet (**Figure 1**), and the trays containing the peat pellets were placed in a low light growth chamber (Percival Scientific Inc., Perry, IA, USA) at 30°C temperature, 100 µmol m^−2^ s^−1^, and 50% relative humidity to promote seed germination. Upon germination, the peat pellets containing the plants were transferred to plant growth chamber (Percival Scientific Inc., Perry, Iowa, USA) which was set to 16-hour day and 8-hour day-night cycle, 33°C temperature, 700 µmol m^−2^ s^−1^, and 50% relative humidity. All trays were watered daily to maintain soil water potential of the plants. The plants were grown to the first vegetative stage (V1), in which soybean plants have 2 nodes, a set of fully developed unifoliate leaves, and a single set of trifoliate leaves that are expanded and fully developed (McWilliams et al., 1999; Purcell et al., 2014). Once the plants reached the V1 stage, the peat was gently washed away from the roots, and the plants were placed in a glass vial containing 10% hydroponic medium in water and allowed to acclimate for 24 hours before testing.

### 2.6 Cell-free culture filtrates

**Figure 2:**
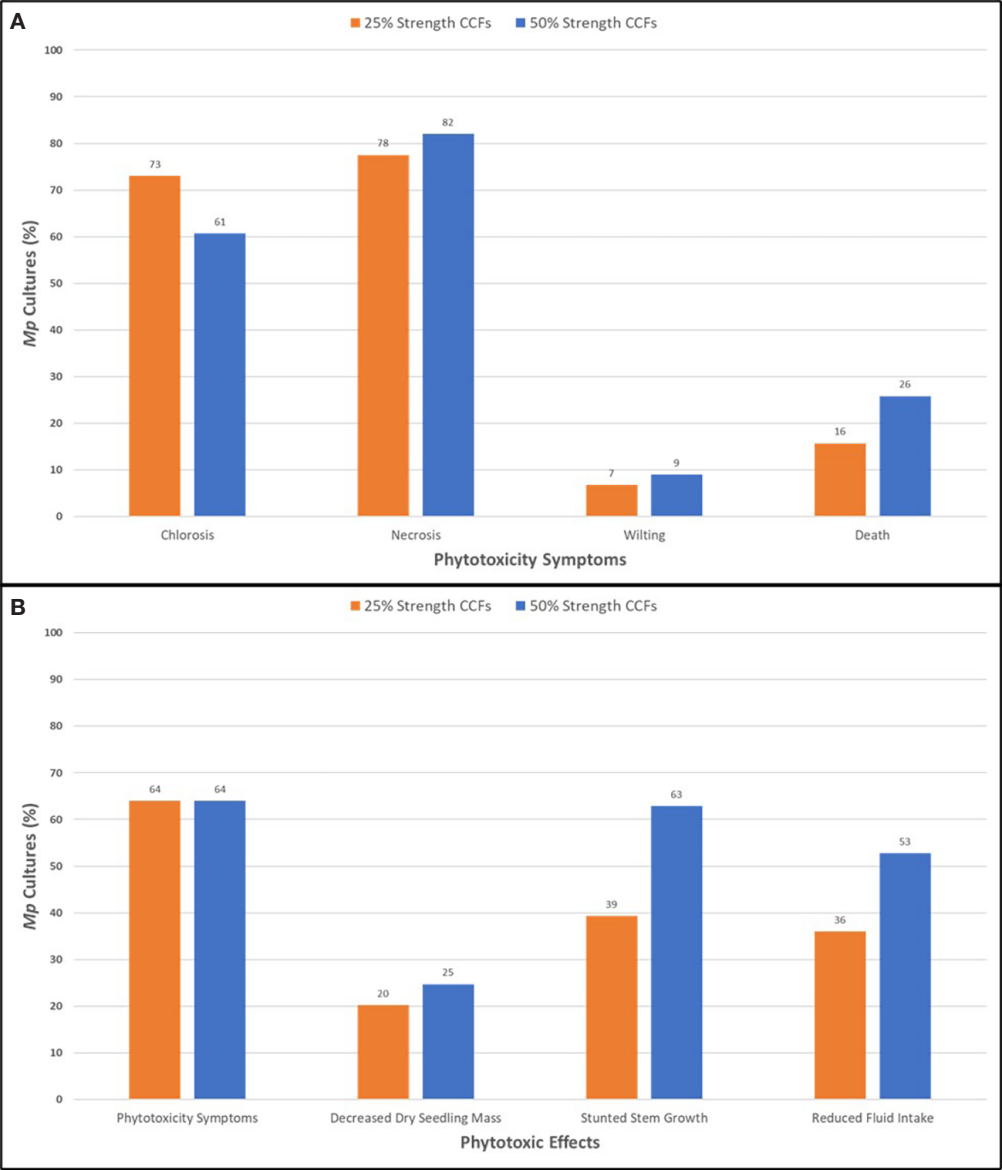
Phytotoxic symptoms **(A)** and effects **(B)** of adding *M. phaseolina* cell-free culture filtrates to the hydroponic culture medium of soybean seedlings of the germplasm line DT97-4290 after 96 hours incubation. *Mp*, *M. phaseolina*; CCF, cell-free culture filtrates.

Mycelial plugs of each *M. phaseolina* isolate were transferred from potato dextrose agar to Czapek-Dox agar and incubated for 5 days in the dark at 28°C. Liquid cultures of *M. phaseolina* were prepared using Czapek-Dox broth, which was prepared by heating 1 L water on a hot plate and stirring in 30 g sucrose, 2.0 g NaNO_3_, 1.0 g K_2_HPO_4_, 0.5 g KCl, 0.5 g MgSO_4_, and 0.01 g FeSO_4_ until dissolved. Once the contents were dissolved, 200 mL Czapek-Dox broth was transferred into 500 mL Erlenmeyer flasks, and the flasks were covered with aluminum foil. The flasks were sterilized for 15 minutes at 121°C and 15 psi in an autoclave (STERIS, Mentor, OH, USA) and cooled to room temperature. Mycelial plugs from 7-day-old Czapek-Dox agar cultures of each isolate were transferred to the flasks. Cotton plugs were fitted to the inoculated flasks, and the flasks were placed on an Innova 40 Benchtop Incubator Shaker (New Brunswick Scientific, Edison, NJ, USA) set to 110 RPM and 28°C and incubated for 10 days (**Figure 2**). After the incubation period, the cultures were initially filtered through cheesecloth to separate out large mycelial growth. The crude filtrates were further purified to cell-free culture filtrates (CCF) by vacuum filtration using sterile, disposable Nalgene Rapid-Flow (Thermo Fisher Scientific, Rochester, NY, USA) filter flasks fitted with 0.45 µm pore-size CN membrane filters. CCFs were stored in freezers at –20°C until use.

### 2.7 Phytotoxicity assays

The phytotoxicity of *M. phaseolina* CCFs against soybean seedlings, germplasm line DT97-4290, in hydroponic growth medium were evaluated in seedlings arranged in a completely randomized design with three replicates per isolate and two dilution levels per isolate. Synthetic (±)-botryodiplodin or cytotoxic culture filtrates were used as positive controls in these and parallel experiments. Each glass vial was randomly assigned a single plant, and each vial represented a replicate. CCFs were diluted with 10% hydroponic medium to 25% strength and 50% strength (v/v) concentrations, and 40 mL scintillation vials were filled with 40 mL diluted CCF. Vials with sterile Czapek-Dox broth diluted to 25% strength and 50% strength (v/v) with 10% hydroponic medium were used as controls. Initial stem length of soybean seedlings was measured when they were placed into vials. The vial openings were plugged with cotton balls, and the vials were placed in a growth chamber with 16-hour day and 8-hour night cycle, 33°C temperature, 700 µmol m^−2^ s^−1^, and 50% relative humidity. After 120 hours, the final stem length and solution remaining in the vial were measured, and symptoms were recorded on a 0-100 scale in increments of 10. Values were assigned based on overall coverage of foliar symptoms such as chlorosis, necrosis, and wilting. The following conversions were used for evaluation: 0 = no symptoms, 10 = green and slight chlorosis, 20 = green and slight-moderate chlorosis, 30 = moderate chlorosis, 40 = moderate-severe chlorosis, 50 = severe chlorosis and slight necrosis (<25%), 60 = slight-moderate necrosis (25%), 70 = moderate necrosis (50%), 80 = moderate-severe necrosis (75%), 90 = severe necrosis (100%), and 100 = complete seedling death (brown and drying). Wilting and death were scored independently as + for wilting or death and – for no observable effect. Values of 1 and 0 were assigned to + and -, respectively for the purpose of statistical analysis. After rating, the seedlings were dried in a laboratory oven (Imperial V Laboratory Oven, Thermo Scientific, Dubuque, IA, USA) at 80°C for 72 hours, and the dry mass of the seedling was weighed using an analytical balance (Mettler Toledo UMX2 Ultra-Microbalance, Mettler-Toledo International, Columbus, Ohio, USA). Evaluation of *M. phaseolina* CCFs at two dilution levels in hydroponic culture medium was essential because soybean seedlings died immediately in 100% CCFs. Also, the Czapek-Dox broth component of the CFFs had a slight phytotoxic effect on soybean seedlings. The range of in-culture phytotoxic responses of soybean seedlings appears to be similar to the range of phytotoxic responses in the field (Meyer et al., 1974; Hartman et al., 2015; Ghosh et al., 2018).

The phytotoxicity of commercially-available mellein against soybean seedlings of the germplasm line DT97-4290 was conducted in 10% hydroponic growth medium and evaluated identically to the CCFs. The assay was arranged in a completely randomized design with three replicates per mellein concentration. A single plant was randomly assigned to each vial, and each vial represented a replicate. A stock solution with 1000 µg/mL concentration was prepared in sterile water with the aid of heating and sonication and diluted with 10% hydroponic medium. The following final concentrations of commercially-available mellein were tested: 0, 1.25, 2.5, 5, 10, 20, 40, 60, 80, and 100 µg/mL. A solution of 10% hydroponic medium containing no mellein served as the negative control for the experiment.

### 2.8 Statistical analysis

All data were statistically analyzed by SAS 9.4 software (SAS Institute, Cary, NC, USA) or the statistical package in Microsoft Excel 2013 (Microsoft Corp., Redmond, WA, USA). Data for soybean seedling stem growth, dry mass, phytotoxicity rating, and growth media volume was analyzed by one-way analysis of variance (ANOVA) for both treatment concentrations. Dunnett’s multiple comparison test at *p* < 0.05, *p* < 0.01, *p* < 0.001, and *p* < 0.0001 level of significance was used to separate the means for each parameter against the control of each treatment set. Correlations assessed for both treatment concentrations with the Pearson correlation coefficient with significance at *p* < 0.05.

## 3 Results

### 3.1 Identification and distribution of mellein in *M. phaseolina* isolates from soybean plants symptomatic of charcoal rot

A total of 89 *M. phaseolina* isolates from soybean plants symptomatic of charcoal rot in the field were grown in Czapek-Dox broth and filtered to produce CCFs. Culture filtrates were analyzed by LC-MS/MS equipped with ESI source, and the secondary metabolites were identified using a custom LC-MS/MS library containing over 900 metabolites (Sulyok et al., 2020). LC-MS/MS analysis of culture filtrates demonstrated that some *M. phaseolina* cultures produced mellein, (-)-botryodiplodin, kojic acid, moniliformin, and other secondary metabolites described previously by Khambhati et al. (2020b).

**Figure 3:**
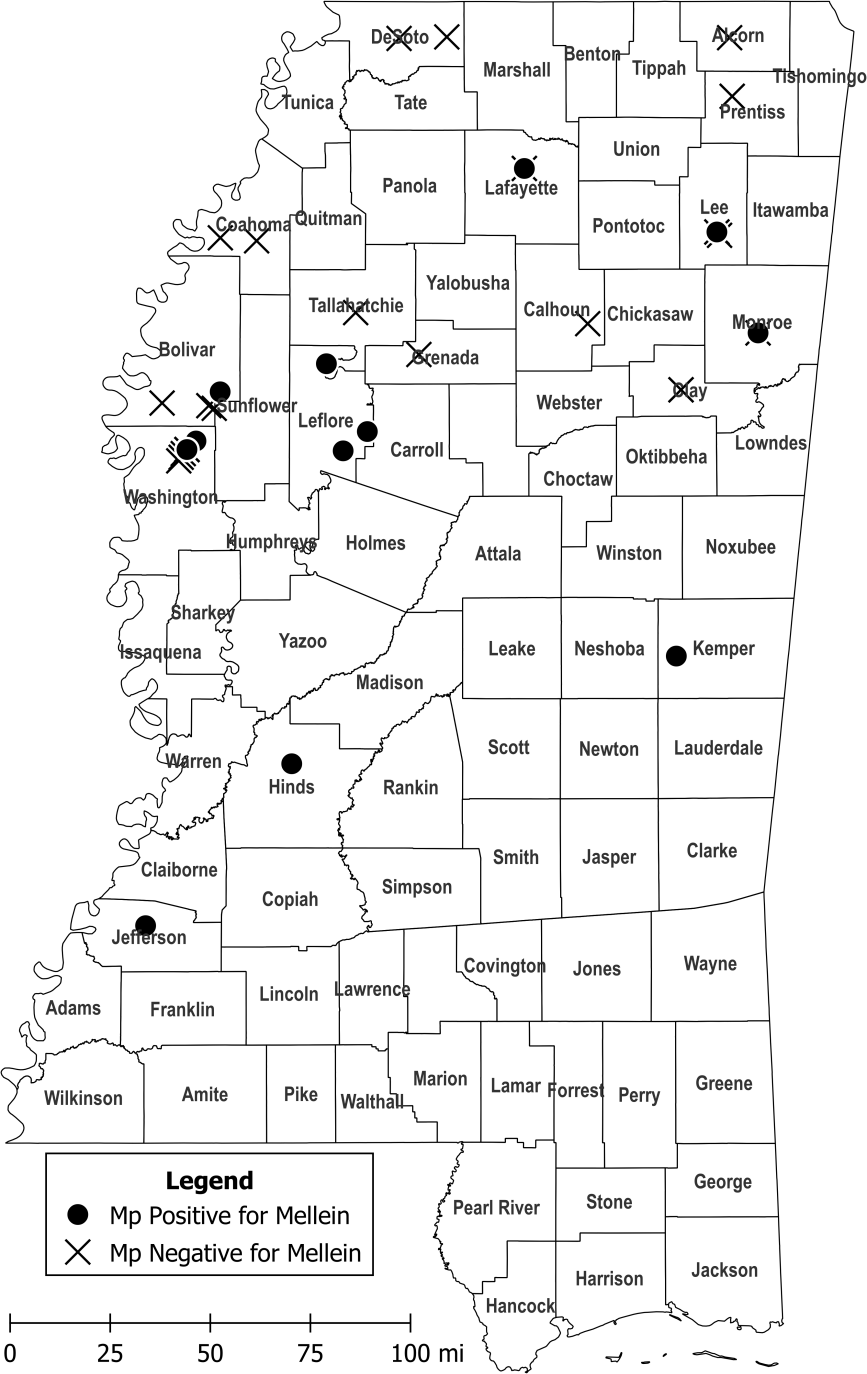
Collection sites of *M. phaseolina* isolates in Mississippi that produced mellein in culture at levels detectable by LC-MS/MS (circles) and collection sites of *M. phaseolina* isolates that did not produce detectable mellein (crosshatches). *M. phaseolina* isolates were also collected from Illinois, Louisiana, Oklahoma, and Tennessee (not shown), but none of them produced mellein in culture. Map figure was created using QGIS, an open-source geographic information system application, and cartographic boundary files available on the US Census Bureau website.

LC-MS/MS analysis showed that 28.1% of *M. phaseolina* isolates produced mellein in liquid culture, and concentrations ranged from 48.9–2203 µg/L with mean concentration of 527.8 µg/L (**Table 1**). All mellein producing isolates originated from Mississippi, but only 37.9% of the Mississippi *M. phaseolina* isolates were found to produce mellein. The mellein-producing isolates were all obtained from the central and southern parts of Mississippi (**Figure 3**) from both Delta and central hill-country soil types. Mellein was not detected by LC-MS/MS in cultures of *M. phaseolina* isolates from other states. The present study also used LC-MS/MS to identify *M. phaseolina* isolates that produced 3-nitropropionic acid (11.5% of isolates studied in the concentration range 5.5 to 554 µg/L), which has not previously been reported to be produced by pathogenic isolates of *M. phaseolina*. All 3-nitropropionic acid producing isolates of *M. phaseolina* were from fields near Jackson, TN.

The frequency of mellein biosynthetic gene clusters in the genomes of *M. phaseolina* isolates was estimated by examining DNA sequences deposited in the National Center for Biotechnology Information (NBCI) GenBank database using antiSMASH, a publicly-available software (Blin et al., 2021). Of nine *M. phaseolina* isolates chosen for completeness of deposited sequences, eight (89%) contained biosynthetic gene clusters for mellein with 100% similarity. All of the eight *M. phaseolina* isolates containing mellein biosynthetic gene clusters were field isolates [from mandarin orange (isolate KE8337) and peach (KE8351) in China; sorghum (MP2) in Australia; jute (MS6) in Bangladesh; strawberry (M11-12), alfalfa (Al-1) and soybean (mp053) in USA; and soybean (mp040) in Paraguay]. The *M. phaseolina* isolate with no mellein biosynthetic gene cluster was from a laboratory isolate [Arabidopsis (Macpha1) in France]. The *M. phaseolina* isolates in this study that do not produce mellein may either lack the biosynthetic gene cluster or the genes are not activated.

### 3.2 Role of mellein in the pathology of *M. phaseolina* in soybean seedlings in hydroponic culture

CCFs from *M. phaseolina* isolates were tested for phytotoxicity by root exposure to soybean seedlings in hydroponic culture (**Figure 1**). The phytotoxic responses to *M. phaseolina* CCFs varied among isolates with symptoms presenting on leaves, stems and roots and ranging from mild chlorosis to complete death. Foliar symptoms included wilting, chlorosis, photobleaching, lesions, necrosis, and vascular discoloration and root symptoms were observed as a pink-red discoloration (**Figure 1**). The phytotoxicity symptoms were distributed as indicated in **Figure 2**. CCFs from 64% of isolates induced phytotoxic symptoms at 50% strength, including 24.7% decreased dry seedling mass, 62.9% stunted stem growth, and 52.8% reduced growth media consumption. CCFs at 25% strength induced phytotoxic symptoms in 64% of isolates, including 20.2% decreased dry seedling mass, 39.3% stunted stem growth, and 36.0% reduced fluid intake (**Table 1** and **Figure 2**). Mellein concentrations measured by LC-MS/MS in CCFs exhibited only very weak, negative, insignificant correlations with several phytotoxicity measures in soybean seedlings following 96 hours of exposure to the roots at 25% (vol/vol) and 50% (vol/vol) concentrations. This observation was made for a combined phytotoxicity rating (at 25% (vol/vol) CCF in culture medium, *r* = -0.133, p = 0.213; at 50% (vol/vol) CCF in culture medium, *r* = -0.118, p = 0.270); for dry weight (at 25% (vol/vol) CCF in culture medium, *r* = -0.110, p = 0.305; at 50% (vol/vol) CCF in culture medium, *r* = -0.117, p = 0.277); for stem growth (at 25% (vol/vol) CCF in culture medium, *r* = -0.103, p = 0.339; at 50% (vol/vol) CCF in culture medium, *r* = -0.117, p = 0.277); for volume of culture fluid absorbed (at 25% (vol/vol) CCF in culture medium, *r* = -0.0367, p = 0.733; at 50% (vol/vol) CCF in culture medium, *r* = -0.135, p = 0.208); for wilting (at 25% (vol/vol) CCF in culture medium, *r* = -0.0358, p = 0.739; at 50% (vol/vol) CCF in culture medium, *r* = -0.0897, p = 0.403); and for death (at 25% (vol/vol) CCF in culture medium, *r* = -0.0399, p = 0.711; at 50% (vol/vol) CCF in culture medium, *r* = -0.103, p = 0.337).

**Figure 4:**
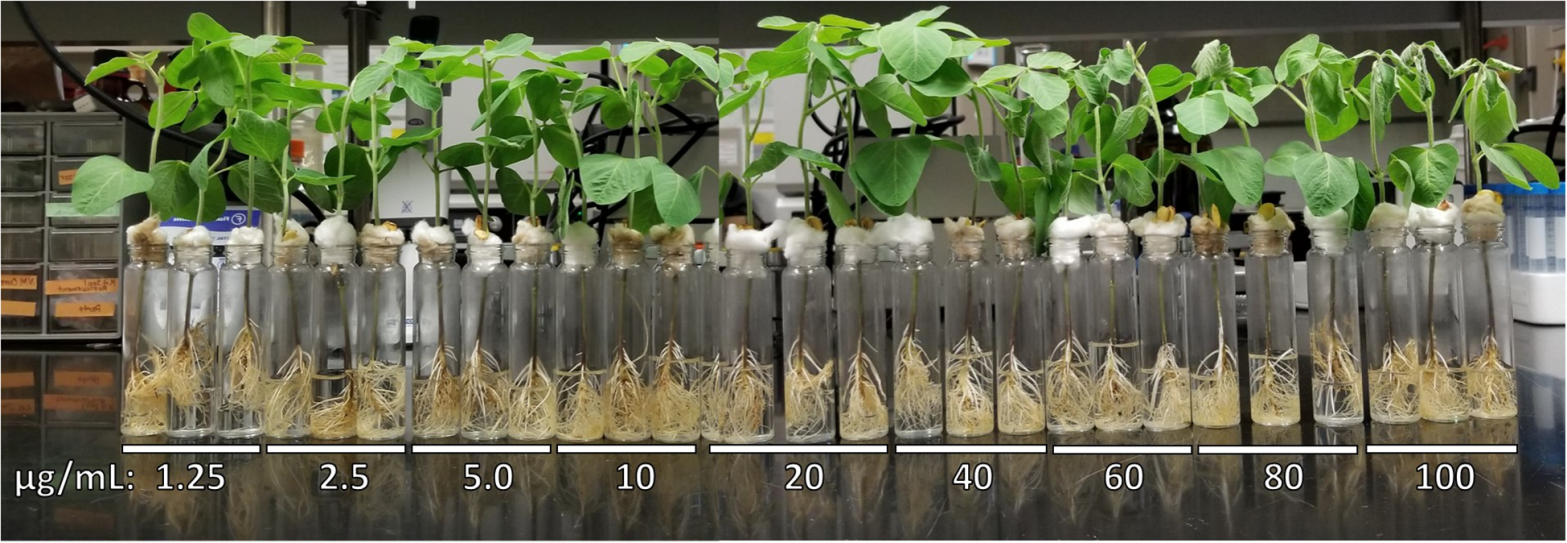
Phytotoxic responses to commercially-available mellein (0-100 µg/mL) against soybean seedlings of germplasm line DT97-4290 after 96 hours in hydroponic culture.

Commercially-available mellein (0-100 µg/mL) added to the hydroponic culture medium of soybean seedlings caused wilting in the concentration range of 40–100 µg/mL as the most prominent phytotoxic effect observed (**Table 3** and **Figure 4**). Wilting was consistently observed only in seedlings treated with the mellein at the highest concentrations tested (40–100 µg/mL) (**Figure 4**), whereas at mellein concentrations below 40 µg/mL, the only symptoms of phytotoxicity observed were reduced uptake of hydroponic culture medium by seedlings (**Table 3**).

**Table 3:** Phytotoxicity of commercially-available mellein against soybean seedlings, germplasm line DT97-4290, in hydroponic culture.

Mellein (µg/mL)	Phytotoxicity Rating^z^	Dry Weight^z^	Stem Growth^z^	Volume Consumed^z^
1.25	NS	NS	NS	NS
2.5	NS	NS	NS	++
5.0	NS	NS	NS	+
10	NS	NS	NS	++
20	NS	NS	NS	NS
40	++++	NS	NS	++
60	++++	NS	NS	++
80	++++	NS	NS	+
100	++++	NS	NS	NS

^z^ Significant differences relative to the control are depicted with ++++ for differences at the *p* < 0.0001 level of significance, as +++ for *p* < 0.001, as ++ *p* < 0.01 and as + for *p* < 0.05. NS denotes no statistical significance.

## 4 Discussion

Analysis by LC-MS/MS of *M. phaseolina* isolates from Illinois, Louisiana, Mississippi, Oklahoma, and Tennessee showed that mellein was produced in culture at varying levels only by isolates originating from Mississippi. Given that different soil types yielded mellein-producing *M. phaseolina* isolates, is not clear what conditions in Mississippi provided a selective advantage for mellein production by *M. phaseolina* there. Production of different mycotoxins in different geographical regions by some fungal species is well established (Awuchi et al., 2021). *M. phaseolina* presumably acquired a biosynthetic gene cluster for mellein originally by genome mining from dead tissue of a fungal or other source. The observation that the biosynthetic gene cluster for mellein could be identified in all field isolates from several widely dispersed countries in the world is consistent with it being acquired early in the evolution of the fungus. The observation in these studies that the majority of *M. phaseolina* isolates did not produce mellein in culture in amounts detectable by LC-MS/MS, is consistent with the biosynthetic gene cluster being either absent, defective or turned off in most isolates. In this study it was observed that *M. phaseolina* isolates producing mellein in culture were located in an east-west band across central Mississippi. Further study is needed to determine if the band extends into neighboring states. Production of mellein in culture by *M. phaseolina* isolates from this region is consistent with the biosynthetic gene cluster for mellein being either acquired, repaired or activated by an unknown process in a *M. phaseolina* strain, and then that strain getting spread throughout the region. Possible mechanisms of spread include riding dust particles on the prevailing west to east winds, or being an endophyte in a cultivar selected for marketing and use at that latitude. The spread of a newly formed mellein-producing strain of *M. phaseolina* would be facilitated if production of mellein provided a selective advantage. Additional studies will be needed to determine the role of mellein in the ecology of *M. phaseolina*. For example, it would useful to know if the mellein biosynthetic gene cluster is present only in the genomes of *M. phaseolina* isolates from central Mississippi that produce mellein in culture, or if mellein biosynthetic gene clusters are present in all or most isolates, regardless of whether they produce mellein in culture or not.

Pathogenic fungi are potentially able to use toxins to facilitate (i) initial infection of plant tissue; (ii) hyphal spread through tissue; (iii) exit from an infected plant in order to spread to adjacent plants; and (iv) penetration of the developing seed in order to establish an endophyte relationship. The ability of mellein to produce localized necrosis in plant tissues suggests the toxin could help a fungus such as *M. phaseolina* accomplish root infection any of those four mechanisms, if the necrotic response is strong enough. However, published studies on the mechanism of action of mellein as a phytotoxin have not focused on mechanisms for producing necrosis on plant tissues. Mellein has been reported to inhibit expression of defense genes in grapes (Ramírez-Suero et al., 2014), elongation of roots and coleoptile in wheat (Bousquet and Skajennikoff, 1974) and reduction of CO_2_ assimilation in wheat seedlings (Bousquet et al., 1977). However, there is no clear relationship of any of these activities to the wilting observed in soybean seedlings in this study.

To determine if mellein production by pathogenic isolates of *M. phaseolina* could play a role in the phytotoxicity observed in soybean seedlings in hydroponic culture, the effect of commercially-available mellein was determined and shown to be wilting at concentrations higher than 40 µg/mL. Cabras et al. (2006) reported similar findings in tomato cuttings with mellein-induced wilting. The phytotoxic response of soybean seedlings to commercially-available mellein added to the culture medium bathing the root occurred at a similar concentration to that reported for apple seedlings (60-100 µg/mL *vs* 150 µg/mL reported for apple), but the toxic response was less intense (wilting vs browning and necrosis for apple) (Li et al., 2021). Given that the threshold concentration for mellein phytotoxicity is 40 times higher than the highest mellein concentration observed by LC-MS/MS in this study, it must be concluded that mellein alone is not responsible for any of the observed phytotoxicity in this study. However, the experiments conducted in this study do not exclude the possibility that mellein may synergize or be additive with other phytotoxic components of the *M. phaseolina* culture fluids examined. Cabras et al. (2006) speculated that 4-hydroxymellein might synergize phytotoxicity (browning, chlorosis and wilting) of tomato cuttings by fungal metabolites in a crude culture fraction, but published evidence for synergy with mellein is lacking. Additional studies will be needed to establish any role for mellein in the infection of soybean by *M. phaseolina*.

## Data availability statement

The raw data supporting the conclusions of this article will be made available by the authors, without undue reservation.

## Author contributions

HA and WS designed the study, secured funding and supervised the work. VK and MS performed the experiments. MS, VK, HA, WS and MT-P contributed to data analysis. VK wrote the first draft of the manuscript and HA and WS edited it. All authors contributed to the article and approved the submitted version.
